# Supplementary material for: Characterization of Immune-Related Molecular Subtypes and a Prognostic Signature Correlating With the Response to Immunotherapy in Patients With Gastric Cancer
Source: Front Immunol. 2022 Jul 8;13:939836. doi: 10.3389/fimmu.2022.939836 (PMC9309259; doi:10.3389/fimmu.2022.939836)
Supplement: Supplementary Table 5 — The results of GO and KEGG enrichment analyses on IDGs. [file DataSheet_1.pdf]

Supplementary Figures

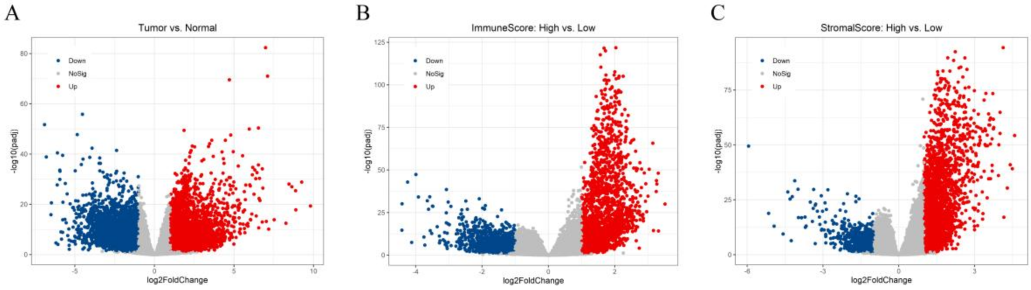

Supplementary Figure 1 | (A-C) Volcano plots of the DEGs in groups of tumors vs. normal tissues **(A)**, high immune scores vs. low immune scores **(B)**, and high stromal scores vs. low stromal scores **(C)**.

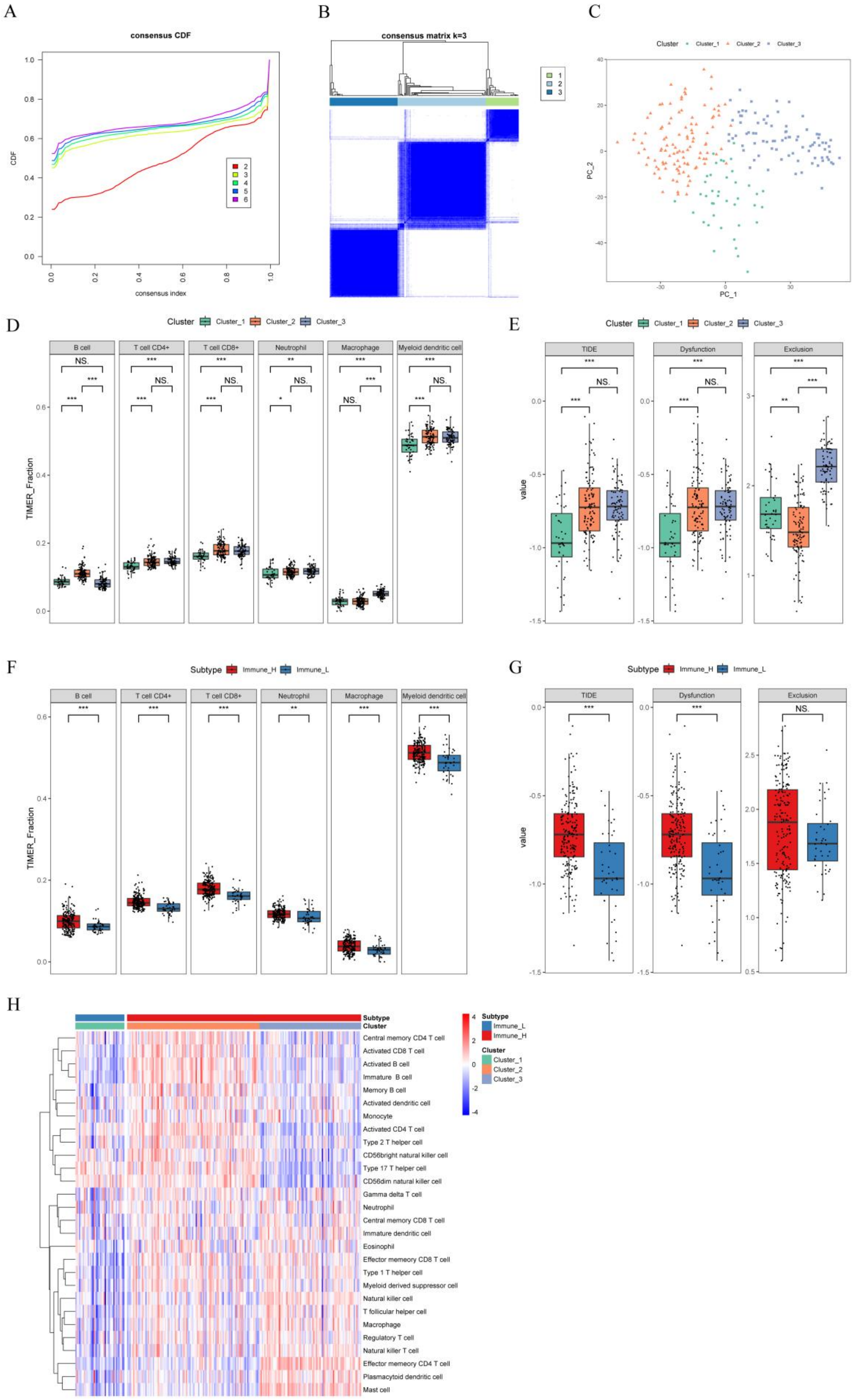
A
consensus CDF
CDF
consensus index
B
consensus matrix k=3
C
Cluster Cluster_1 Cluster_2 Cluster_3
PC_1
PC_2
D
Cluster Cluster_1 Cluster_2 Cluster_3
B cell
T cell CD4+
T cell CD8+
Neutrophil
Macrophage
Myeloid dendritic cell
TIMER_Fraction
E
TIDE
Dysfunction
Exclusion
value
F
Subtype Immune_H Immune_L
G
H
Subtype
Cluster
Immune_L
Immune_H
Cluster_1
Cluster_2
Cluster_3
Central memory CD4 T cell
Activated CD8 T cell
Activated B cell
Immature B cell
Memory B cell
Activated dendritic cell
Monocyte
Activated CD4 T cell
Type 2 T helper cell
CD56bright natural killer cell
Type 17 T helper cell
CD56dim natural killer cell
Gamma delta T cell
Neutrophil
Central memory CD8 T cell
Immature dendritic cell
Eosinophil
Effector memeory CD8 T cell
Type 1 T helper cell
Myeloid derived suppressor cell
Natural killer cell
T follicular helper cell
Macrophage
Regulatory T cell
Natural killer T cell
Effector memeory CD4 T cell
Plasmacytoid dendritic cell
Mast cell

Supplementary Figure 2 | Reproduction of immune-related clusters and subtypes in the HMU-GC cohort. **(A)** The cumulative distribution function (CDF) curves for k = 2 to 6. **(B)** Three clusters were identified through consensus clustering. **(C)** PCA analysis revealed the dissimilarity among three clusters. **(D, E)** The fractions of tumor-infiltrating cells estimated by TIMER **(D)** and TIDE scores **(E)** among three clusters. **(F, G)** The fractions of tumor-infiltrating cells estimated by TIMER **(F)** and TIDE scores **(G)** between the two immune subtypes. **(H)** The levels of 28 immune cells of GC samples in the two immune subtypes were calculated through ssGSEA. NS. or ns: no statistical significance, *$p < 0.05$, **$p < 0.01$, ***$p < 0.001$.

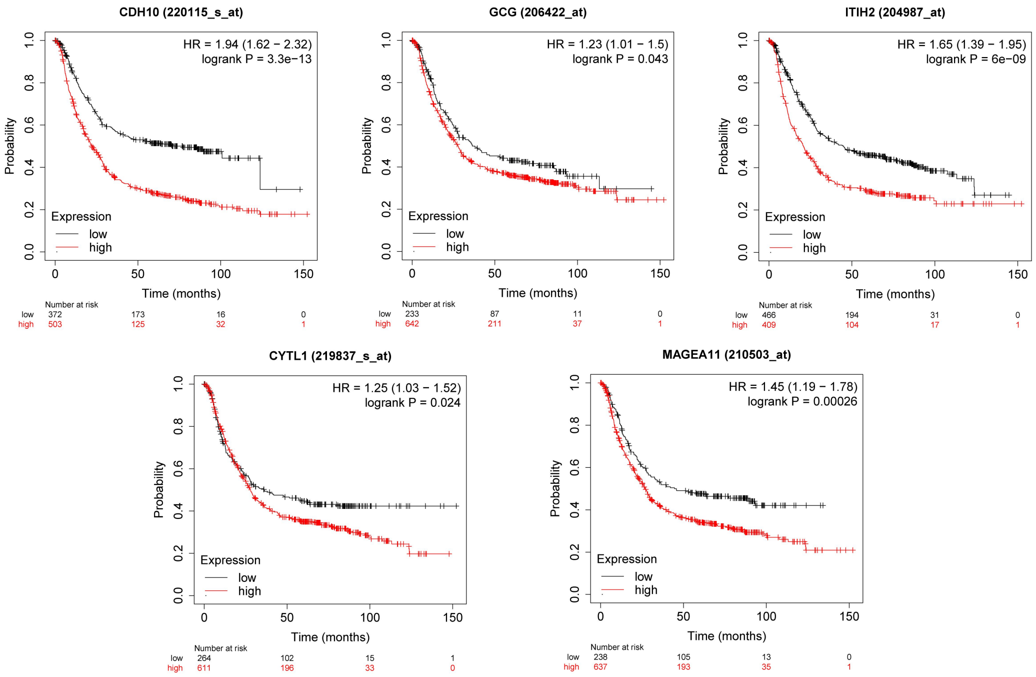

Supplementary Figure 3 | The results from Kaplan-Meier Plotter confirmed the prognostic value of the five selected genes.

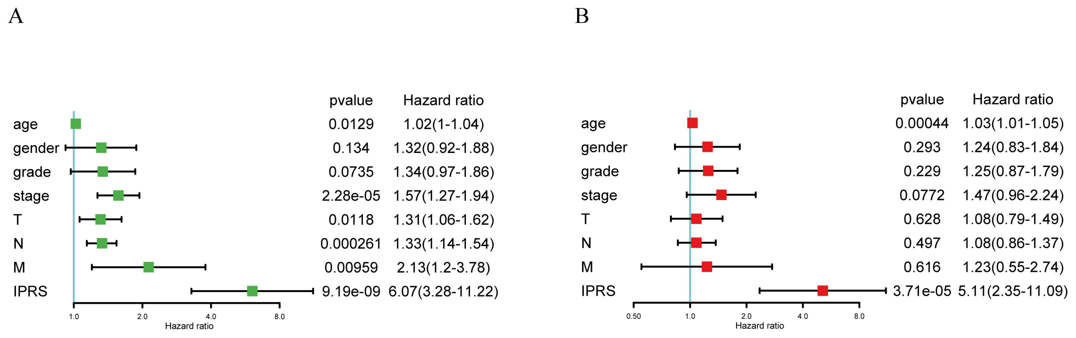

Supplementary Figure 4 | (A, B) Univariate **(A)** and multivariate **(B)** Cox regression analyses of IPRS and clinicopathological features.

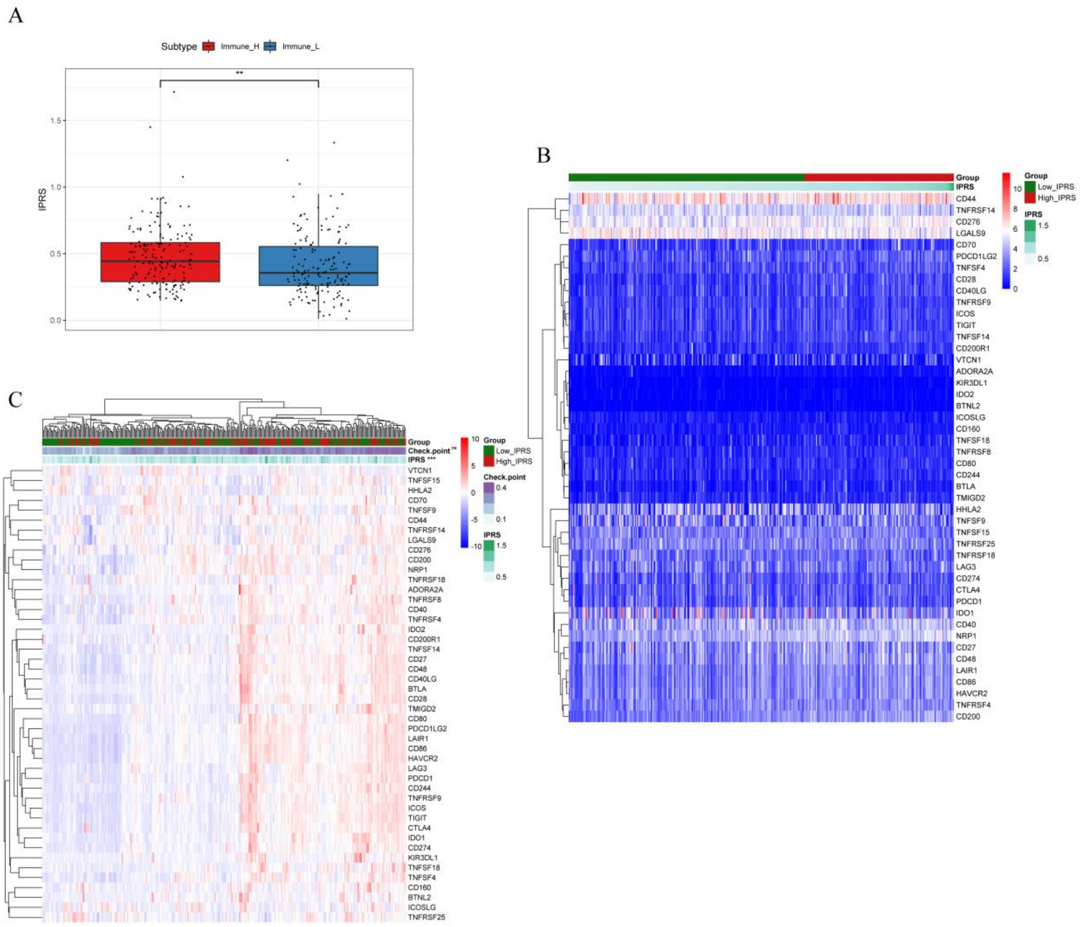

Supplementary Figure 5 | (A) The relationships between IPRS and immune subtypes. **(B)** The heatmap portraying the relationships between IPRS and immune checkpoints with the original gene expression value. **(C)** The heatmap portraying the relationships between IPRS and immune checkpoints with the gene expression value scaled by row.

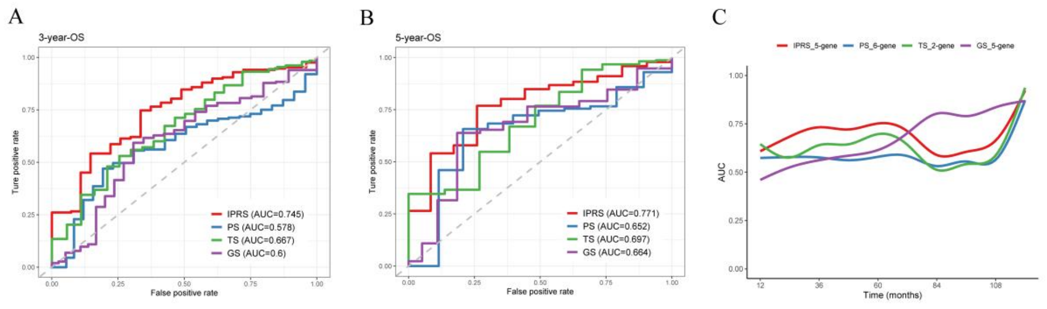

Supplementary Figure 6 | Comparison of the efficacy of IPRS and other prognostic signatures. **(A, B)** The ROC curves of four prognostic signatures at 3 years **(A)** and 5 years **(B)**. **(C)** The time-dependent AUC curves of four prognostic signatures. PS: pyroptosis-related signature; TS: two-gene signature; GS: glycolysis-related signature.

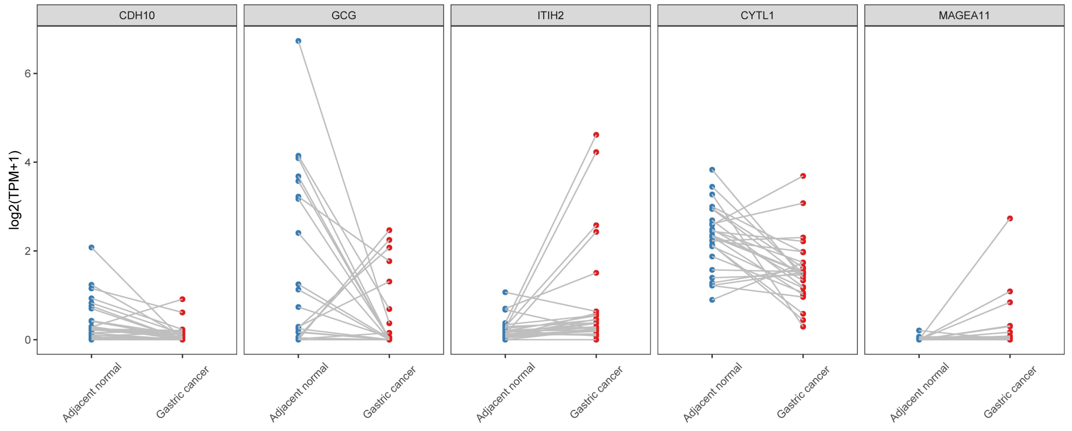

Supplementary Figure 7 | Analyses of the paired GC samples and adjacent normal tissues.
